# Why Do Psychiatrists Still Prescribe Valproate to Women of Childbearing Potential?

**DOI:** 10.3389/fpsyt.2020.00739

**Published:** 2020-07-24

**Authors:** Aleksei Rakitin

**Affiliations:** Department of Neurology and Neurosurgery, University of Tartu, Tartu, Estonia

**Keywords:** congenital malformation, valproate, valproat sodium, valproate (CID 3121), teratogenicity, fetal abnormalities

## Introduction

Valproic acid (VPA) is one of the most frequently prescribed anti-seizure medications (ASMs) (1). VPA has the widest spectrum of use among ASMs, being effective against all seizure types at an efficacy comparable to those of alternative ASMs. VPA is especially effective against absence seizures and juvenile myoclonic epilepsy (2). This drug is used widely in psychiatric practice, mainly in the treatment of acute episodes of bipolar disorder and for episode prophylaxis (3).

## Teratogenicity of Valproate

The first observations of teratogenic effects of VPA were reported in the early 1980s (4, 5). Since that time, analyses of data from different pregnancy registries have consistently shown that maternal VPA use during pregnancy increases the risk of birth defects in fetuses and infants (6–8). VPA use during the first trimester of pregnancy is related to increased risks of spina bifida, craniosynostosis, cleft palate, hypospadias, atrial septal defects, and polydactyly. The risks of teratogenic side effects are generally two to three times greater for VPA than for other ASM monotherapies (9). The risk of fetal malformations associated with maternal polytherapy depends mainly on whether the regimen includes VPA (10, 11). Recent reports emphasize that the teratogenic effect of VPA is dose related; the risk of major congenital malformations associated with VPA doses < 700 mg/day seems to be comparable to those associated with other ASMs (12). There are reports of impaired intellectual and behavioral development among children who were exposed to VPA *in utero*. School-aged children of mothers who were treated with VPA during pregnancy are at increased risk of having lower verbal intelligence quotient scores than are children exposed to other ASMs, such as carbamazepine and phenytoin, and those with no ASM exposure (13). Studies have also demonstrated associations between fetal VPA exposure and the risks of autism and autism spectrum disorder (14). In addition, data from the Neurodevelopmental Effects of Antiepileptic Drugs study suggest that fetal VPA exposure is related to attention deficit hyperactivity disorder (15).

The precise molecular mechanism of VPA teratogenicity is unknown. VPA increases the formation of reactive oxygen species and induces apoptosis in post-implantation embryos (16), and it inhibits vascular endothelial growth factor expression and activity (17). It alters the Wnt and mTOR signaling pathways, which play important roles in the pathogenesis of autism spectrum disorder (18). However, the main cause of teratogenicity is probably VPA’s promotion of histone acetylation *via* histone deacetylase (HDAC) inhibition. HDAC inhibition could affect the expression levels of 2%–5% of all genes (19). This characteristic of VPA appears to have significant impacts on embryogenesis (20), with an increased rate of teratogenic side effects among children exposed to this medication *in utero* (21).

## Guidelines

Accumulating evidence regarding the teratogenicity of VPA has led to discussions in the medical and scientific communities in the last decade, which have resulted in regulatory agency actions. In 2013, the US Food and Drug Administration recommended not to use of VPA-based prescription to pregnant women and those of childbearing potential (22). In 2014, the European Medicines Agency (EMA) strengthened its warnings regarding VPA use by females. The agency proposed that, when treatment with VPA was unavoidable, women of childbearing potential should use effective contraception and VPA treatment should be managed by doctors experienced in treating the corresponding conditions. Thereafter, the Commission on European Affairs of the International League Against Epilepsy (CEA-ILAE) and the European Academy of Neurology (EAN) developed specific recommendations for the avoidance of VPA use in women of childbearing potential and VPA prescription as a first-line treatment for focal epilepsy. For epilepsy syndromes that are treated most effectively with VPA, the recommendations specify that this drug be offered to females only after risk–benefit assessment and discussion with the patient and/or her representatives. When possible, VPA doses not exceeding 500–600 mg/day are recommended, although higher doses may be used to achieve seizure freedom (23). After French national data showed unsatisfactory effects of the restrictions established in 2014 (24), the EMA adopted a new procedure that resulted in the publication of renewed restrictive measures in May 2018 (25, 26). The EMA prohibited the use of VPA for bipolar disorder and migraine, as well as VPA use for epilepsy during pregnancy, unless no alternative effective treatment was available. Many other national medicine agencies worldwide have issued similar recommendations in recent years; generally, VPA prescription to young females is allowed only in cases of diagnosed epilepsy in which seizures cannot be controlled by other ASMs (27–29).

## The Impact of Regulatory Restrictions on Valproate Prescriptions

During the last decade, several papers on the impacts of these restrictions on VPA prescription patterns were published. One of the first studies addressing this issue was conducted in Ireland, where VPA prescription trends were assessed during the period of 2008–2013. The rate of VPA prescription to women aged 15–44 years declined slightly from 3.5/1,000 eligible population in 2008 to 3.14/1,000 in 2013. This declining trend was more obvious for women with epilepsy, but VPA prescription rates for other indications increased (30). A report from the UK showed a significant increase in VPA prescription to young females with bipolar disorder from 1995 to 2007 (31). Similarly, US data on individual prescriptions given during patients’ visits to office-based physicians and outpatient clinics in 1996–2007 showed that 83% of VPA prescriptions were issued to females without epilepsy, and that 74% of these prescriptions were for psychiatric diagnoses. The rate of ASM prescription among women without epilepsy increased more than three times during the study period, whereas the VPA prescription rate remained relatively stable (32). In a Florida Medicaid study, despite the overall decline in VPA use among pregnant women, the use of VPA for psychiatric disorders remained stable (33). Data from the Australian Register of ASMs in Pregnancy for the period 1999–2007 showed that VPA prescription to pregnant women decreased over time, but that VPA prescription trends in the general population increased, possibly related to the expanded use of the drug for psychiatric indications, especially bipolar disorder (34). More recent data from Sweden showed that the 2014 regulation significantly influenced the rate of VPA prescription to young females with psychiatric diagnoses. A comparable decline in the rate of VPA prescription to females with epilepsy began earlier, suggesting that psychiatrists became aware of VPA-associated fetal risks significantly later than did neurologists. (35). A study from Estonia shows a similar phenomenon; the increasing trend in VPA utilization among reproductive-aged women ended after the 2014 publication of the EMA restrictions, due mainly to declines in neurologists’ prescription rates. However, this regulatory did not changed VPA prescription rates among psychiatrists (36). Despite the availability of a massive amount of information about VPA-related fetal risks, psychiatric disorders remain the indications prompting about 40%–50% (or even more) of prescriptions of the drug to young females in many countries (32, 35–38). This phenomenon is incomprehensible and disturbing, as the drug is most effective in subgroups of patients with epilepsy (e.g., those with juvenile myoclonic epilepsy) and cannot be replaced among these patients without the risk of a significant increase in seizure frequency. However, no such subgroup of psychiatric patients is known; VPA can be replaced by lithium or antipsychotics for these patients. A meta-analysis of randomized control trials showed that VPA was probably less effective than haloperidol, olanzapine, and quetiapine as monotherapy for patients with acute mania episodes (39). In the treatment of bipolar depression, quetiapine, lurasidone, lithium, and lamotrigine in combination with selective serotonin reuptake inhibitors are at least as effective as VPA (40–42). In addition, lamotrigine is known to have one of the lowest teratogenic potentials among studied ASMs (43).

## Discussion

So, why do psychiatrists still prescribe VPA for the treatment of psychiatric disorders in women of childbearing potential? The reasons for such prescription are not clear, but several potential explanations can be offered. The discussion about risks associated with fetal VPA exposure has proceeded mainly in epilepsy-related medical journals, which are read by neurologists. Epileptologists did not agree with some of the recommendations included in the 2014 EMA warning. For example, the EMA recommended trying less-appropriate ASMs before VPA prescription to women with VPA-responsive epileptic syndrome. It also advised doctors to “consider treatment alternatives if a female becomes pregnant.” This recommendation could lead to attempts to replace VPA with other medications, which could result in increased seizure frequency during pregnancy. As a result of these concerns, the CEA-ILAE and EAN developed specific recommendations for VPA use by young females with epilepsy, which was covered widely in neurology-related journals and at international neurological meetings (23). Psychiatrists did not participate in this discussion; psychiatric journals contain only sporadic articles addressing this issue (3, 38, 44, 45). Another reason could be the inconsistency of recommendations in available guidelines. Although information about VPA-associated fetal risks exists and important advice is available, some guidelines lack a set of mandatory and binding rules. Some guidelines call for the discussion of VPA-associated risks with patients and attention to contraceptive and folic acid use (38), whereas others, such as those of The National Institute for Health and Care Excellence, states that “VPA must not be used in women and girls of childbearing potential (including young girls who are likely to require treatment into their childbearing years), unless other options are unsuitable and a pregnancy prevention program is in place” (46). The majority of guidelines related to VPA-associated fetal risks focus on the treatment of women with epilepsy, explaining the greater awareness of these risks in the neurological community and lack of awareness among psychiatrists (38).

To conclude, medical specialists should be aware of the wide range of risks associated with VPA prescription to young females, especially when a broad range of alternative treatments is available. Relevant organizations should intensify their efforts to develop and implement strict and unified interdisciplinary guidelines regarding VPA use in women of childbearing potential. In addition, more efforts should focus on informing psychiatrists about VPA-related fetal risks.

## Author Contributions

The author confirms being the sole contributor of this work and has approved it for publication.

## Conflict of Interest

The author declares that the research was conducted in the absence of any commercial or financial relationships that could be construed as a potential conflict of interest.
